# Ultra-processed food consumption and its correlates among Italian children, adolescents and adults from the Italian Nutrition & Health Survey (INHES) cohort study

**DOI:** 10.1017/S1368980021002767

**Published:** 2021-06-24

**Authors:** Emilia Ruggiero, Simona Esposito, Simona Costanzo, Augusto Di Castelnuovo, Chiara Cerletti, Maria Benedetta Donati, Giovanni de Gaetano, Licia Iacoviello, Marialaura Bonaccio

**Affiliations:** 1 Department of Epidemiology and Prevention, IRCCS NEUROMED, Via dell’Elettronica, 86077 Pozzilli (IS), Italy; 2 Mediterranea Cardiocentro, Napoli, Italy; 3 Department of Medicine and Surgery, Research Center in Epidemiology and Preventive Medicine (EPIMED), University of Insubria, Varese-Como, Italy

**Keywords:** Ultra-processed food, General population, Socio-economic factors, Psychosocial factors

## Abstract

**Objective:**

To assess ultra-processed food (UPF) consumption and its socio-demographic, psychosocial and behavioural correlates in a general population of Italian children, adolescents and adults.

**Design:**

Cross-sectional telephone-based survey

**Setting:**

Italy, 2010–2013.

**Participants:**

In total, 9078 participants (5–97 years) from the Italian Nutrition & Health Survey. Dietary intakes were collected by a 1-d 24-h dietary recall. UPF was defined by the NOVA classification and expressed as percentage of total energies.

**Results:**

Average energy intake from UPF (95 % CI) was 17·3 % (17·1 %, 17·6 %) among adults and 25·9 % (24·8 %, 27·0 %) in children/adolescents. Top sources of UPF were processed meats (32·5 %) and bread substitutes (16·7 %). Among adults, age (*β* = −3·10; 95 % CI (−4·40, −1·80) for >65 years *v*. 20–40 years; *β*s are dimensionless) and residing in Southern Italy (*β* = −0·73; 95 % CI (−1·32, −0·14) *v*. Northern) inversely associated with UPF. Screen view during meals was directly linked to UPF, as well as poor self-rated health (*β* = 5·32; 95 % CI (2·66, 7·99)), adverse life events (*β* = 2·33; 95 % CI (1·48, 3·18)) and low sleep quality (*β* = 2·34; 95 % CI (1·45, 3·23)). Boys consumed two-point percent more UPF of the total energy than girls (*β* = 2·01; 95 % CI (0·20, 3·82)). For all ages, a Mediterranean diet was inversely associated with UPF (*β* = −4·86; 95 % CI (−5·53, −4·20) for good *v*. poor adherence in adults and (*β* = −5·08; 95 % CI (−8·38, −1·77) for kids).

**Conclusions:**

UPF contributes a modest proportion of energy to the diets of Italian adults while being one-quarter of the total energies in children/adolescents. UPF was associated with several psychosocial factors and eating behaviours. Increased adherence to Mediterranean diet would possibly result in lower UPF consumption.

Ultra-processed food (UPF) is made by industrial processing or chemical synthesis, from processed substances extracted or refined from whole foods, and they are rich in additives used to imitate or enhance the sensory features of foods, such as colour stabilisers, flavour enhancers and non-sugar sweeteners^(1–3)^. UPF is generally poor in micronutrient and fibres, but rich in fats, added sugar, salt and energy, and their packaging could be harmful to health^(2)^. The classification system NOVA (a name, not an acronym) is widely used at epidemiological level and rates foods according to the extent and purpose of processing into one of the following categories: (1) unprocessed or minimally processed foods; (2) processed culinary ingredients, processed foods and (3) UPF and drink products^(4)^. Excessive consumption of UPF has been associated with higher risk of metabolic conditions predisposing to increased health risk, such as obesity^(5)^, hyperlipidaemia^(6)^, hypertension^(7)^, diabetes^(8)^ and metabolic syndrome^(9)^.

Also, a numerous large prospective studies^(10–13)^ and meta-analyses^(14,15)^ provided evidence that a larger share of UPF in the diet leads to a rise in the risk of diet-related chronic disease.

The health impact of food processing has become a relevant and timely topic given the increasing volume of industrially processed food worldwide. Processed food constitutes a large part of the world’s food consumption and is remarkably high in non-Mediterranean countries, representing almost 60 % of total energies in the USA^(16)^ and in the UK^(17)^, 42 % in Australia^(18)^ and 46 % in Canada^(19)^, with differences between adults and children/adolescents.

In a Mediterranean country such as Spain, the proportion of food that is ultra-processed is about 24 %^(20)^, possibly because home cooking is part of a Mediterranean diet^(20)^.

Despite the mounting epidemiological evidence at international level associating higher dietary share of UPF intake with adverse health outcomes, there is a lack of data in Italy regarding UPF consumption and also differences among socio-demographic strata, a major deficiency for developing effective public health policies.

Italy has been long characterised by a Mediterranean diet, the traditional diet of the olive tree-growing areas of the Mediterranean Sea that features whole or minimally processed foods and emphasises food preparation^(21)^. The only available estimates for consumption of UPF in Italy derive from national household budget surveys collected in 1996 showing an average dietary share of UPF of 13·4 %^(22)^, but individual-level consumption data are lacking. Quite recently, an analysis from the Moli-sani cohort estimated UPF consumption in the adult population of Molise, but data on children/adolescents were not available^(13)^.

To fill this knowledge gap, the aim of the present study was to describe the intake of UPF in Italian adults and children and to identify its main predictors. We thought that the traditionally high adherence of Italians to the Mediterranean diet might be associated to lower consumption of UPF, as compared with non-Mediterranean countries.

We took advantage of data collected by the Italian Nutrition & Health Survey (INHES study), a telephone-based survey conducted throughout Italy in 2010–2013 on 9139 participants aged 5–97 years.

## Methods

### Study population

The INHES cohort study is a 3-year telephone-based survey on nutrition and health specifically designed to collect information on dietary habits (quality, quantity and food patterns), food choice determinants and food health awareness of the Italian population according to geographical distribution, age, gender and socio-economic profile.

Between November 2010 and November 2013, 9319 men and women aged ≥5 years from all over Italy were enrolled. Details about this cohort have been previously described^(23)^.

Briefly, 9106 subjects in the age range 35–79 years, recruited in the 2008–2012 wave of the Italian Cardiovascular Epidemiologic Observatory^(24)^ (participation rate 53 %, from 40 % to 85 % in the different regions), were invited to participate in the INHES survey. Once they accepted, participants were asked to invite one relative older than 79 or younger than 35 years to join the survey.

Finally, 5385 (59·1 %) from the original population and 3754 from their relatives were included in the survey for a total of 9139 participants.

The sampled subjects were distributed across four seasons (excluding Christmas, Easter and mid-August periods), and the survey calendar was organised to capture an adequate proportion of weekdays and weekend days at group level.

The recruitment was performed using computer-assisted telephone interviewing (CATI); data on diet and dietary-related behaviors, health status, common risk factors, anthropometry and health perception were collected.

For the purpose of the present study, we excluded subjects with missing values for BMI (0·2 %), smoking habits (0·4 %), socio-economic variables (occupation, education and marital status; 0·8 %) or reporting implausible energy intakes (<800 kcal/d in men and <500 kcal/d in women or >4000 kcal/d in men and >3500 kcal/d in women; 2·3 %). Finally, a total of 9078 subjects were included in the analyses.

### Dietary assessment

Data on food intake were collected through a self-recorded diary, by using a computer-based 1-d 24-h dietary recall interview (24-HDR) software, and the Italian version of the European Food Propensity Questionnaire was also administered^(25,26)^.

For every eating occasion, subjects were asked to carefully record and recall: (a) time and place of consumption; (b) detailed description of foods (or beverages) and (c) quantity consumed and brand (for manufactured foods). Portion sizes were reported by subjects with the help of a picture booklet. Moreover, participants were asked if they were on a particular diet and if the consumption they had reported differed from their usual one.

Participants’ food consumption of single food items or recipes was ‘translated’ by the nutrition specialist during the interview into food items or recipes included in the food list of the data management system INRAN-DIARIO 3·1^(25,27)^.

The final output database included information for the daily consumption of the 2000 single food items that were included in the software food list.

We used the NOVA classification^(4)^ to categorise each food item into one of the following categories according to the extent and purpose of food processing: (1) fresh or minimally processed foods (e.g. fruits and vegetables, meat and fish); (2) processed culinary ingredients (e.g. honey and butter); (3) processed foods with salt, sugar or oil (e.g. canned or bottled vegetables and legumes, canned fish) and (4) UPF containing predominantly industrial substances and little or no whole food (e.g. carbonated drinks and processed meat). For the purpose of this study, we used the latter NOVA group. We ultimately identified a total of twenty-five food groups that fell into the UPF category according to NOVA (see online Supplemental Table 1).

To calculate the proportion of energy from each group of the NOVA classification, we divided the energy content of each group by total energy intake. Quartiles of energy intake from UPF were also generated for analysis purposes.

Adherence to the Mediterranean diet in adults was evaluated by the Mediterranean Diet Score (MDS) as proposed by Trichopoulou *et al.* ^(28)^ and categorised into tertiles as good (MDS ≥ 5 points), average (4 points) and poor adherence (0–3 points). Adherence to the Mediterranean diet in children and adolescents was evaluated by the KIDMED index (Mediterranean Diet Quality Index) for children and teenagers^(29)^, classified as follows: good (≥6 points, indicating an optimal adhesion to Mediterranean diet); average (4–5 points) and poor (≤3 points).

### Meal patterns and eating behaviours

Meal patterns comprised both patterning of main meals (breakfast, lunch and dinner) and context of main meals, such as meals eaten out of the home, or time of consumption or eating meals in front of the television or when using PC. Information on daily amount of time spent in watching TV/using PC was also collected.

### Socio-economic and psychosocial factors

Education was based on the highest qualification attained and was categorised as up to elementary school (corresponding to ≤ 5 years of study), lower secondary (>5 ≤ 8 years), upper secondary (>8 ≤ 13 years) and post-secondary (>13 years).

Present occupation was assembled into the following six groups: manual, non-manual, housewife, retired, student and unemployed. Marital status was defined as married/living in a couple, unmarried, separated/divorced and widowed.

Self-rated health was assessed through one-item question (‘in general, how would you rate your health status’), and responses were arranged along a four item Likert-type scale from ‘excellent’ to ‘poor’.

Information on psychosocial conditions during the previous 12 months were gathered by administering a standard set of questions^(30)^.

Major adverse life events (yes/no) were assessed by asking participants whether in the past year they had experienced one or more of the following: (1) marital separation or divorce; (2) business failure; (3) major intra-family conflict; (4) death or major illness of a close family member; (5) loss of job or retirement, violence; (6) death of a spouse; (7) major personal injury or illness or (8) other major stress.

Psychological stress was assessed through two single-item questions relating to stress at work and home by asking participants how often in the past year they had felt stressed by indicating one of the following response options: (1) never; (2) sometimes; (3) most of the times; (4) often and (5) always.

Level of financial stress was defined as (1) little or none; (2) moderate or (3) high.

Perceived control on the job-related activities was assessed by asking participants to rate their level of autonomy in organising their own working days as (1) none; (2) little; (3) moderate; (4) good; (5) very good and (6) not currently working.

Finally, sleep quality was assessed through a single item question: ‘In general, how would you rate your sleep?’ with response options being: (a) restless and (b) restful.

### Assessment of covariates

Urban or rural environments were defined on the basis of the urbanisation level as described by the European Institute of Statistics (EUROSTAT definition) and obtained by the tool ‘Atlante Statistico dei Comuni’ provided by the Italian National Institute of Statistics^(31)^.

Subjects were classified as never (who has never smoked or who has smoked less than 100 cigarettes in the lifetime), current (smoking one or more cigarettes/d at the time of interview), former (who had quit smoking at the time of interview) or occasional smokers (smoking less than 1 cigarette/d at the time of interview).

History of CVD and cancer and previous diagnosis of diabetes, hypercholesterolaemia and hypertension was self-reported and categorised as no/yes.

In adults, BMI was calculated by using self-reported measurements of height and weight, calculated as kg/m^2^ and grouped into three categories as normal (≤25 kg/m^2^), overweight (>25 < 30 kg/m^2^) or obese (≥30 kg/m^2^). BMI in children/adolescents was categorised according to specific values for children considering sex and age^(32)^. Sport activity was self-reported and used as a dichotomous variable (yes/no).

### Statistical analysis

Main characteristics of the study population are presented as numbers and percentages for categorical values and means with sd for continuous variables (Table 1).

**Table 1:** Characteristics of participants from the INHES study cohort, Italy 2010–2013

	Adults (20–97 years)		Children/adolescents (5–19 years)	
	*n*	%	*n*	%
*N* of subjects	8569	100·0	509	100·0
Total energy (kcal/d)				
Mean	1926·4		2081·7	
sd	588·3		653·6	
% Energy from UPF				
Mean	17·3		25·9	
95 % CI	17·1, 17·6		24·8, 27·0	
% Energy from processed food				
Mean	24·4		18·6	
95 % CI	24·1, 24·7		17·6, 19·6	
% Energy from culinary ingredients				
Mean	19·6		17·4	
95 % CI	19·5, 19·8		16·8, 18·0	
% Energy from unprocessed food				
Mean	38·7		38·1	
95 % CI	38·4, 38·9		37·2, 39·1	
Men (adults)/boys	3977	46·4	261	51·3
Age, years				
Mean	56·9		14·5	
sd	14·6		3·7	
Geographical area				
Northern Italy	3500	40·9	111	21·8
Central Italy	1391	16·2	44	8·6
Southern Italy	3781	42·9	354	69·6
Place of residence				
Rural	1168	13·6	66	13·0
Urban	7401	86·4	443	87·0
Educational level				
Up to elementary	1523	17·8	185	36·4
Lower secondary	2258	26·4	320	55·9
Upper secondary	3413	39·8	4	0·8
Post-secondary	1375	16·0	–	
Occupation				
Manual	1521	17·8	–	
Non-manual	2637	30·8	1	0·2
Housewife	952	11·1	–	
Retired	3071	35·8	–	
Student	140	1·6	503	98·8
Unemployed	248	2·9	5	1·0
Marital status				
Married/in couple	6470	75·5	3	0·6
Unmarried	1221	14·2	506	99·4
Separated/divorced	272	3·2	–	
Widowed	606	7·1	–	
Smoking habit				
Non-smoker	5123	59·8	451	88·6
Current	1374	16·0	40	7·9
Former	1909	22·3	3	0·6
Occasional	163	1·9	15	2·9
Sport activity	1559	18·2	337	66·2
CVD	289	3·4	0	0·0
Cancer	286	3·3	1	0·2
Hypertension	2762	32·2	2	0·4
Hyperlipidaemia	1888	22·0	6	1·2
Diabetes	644	7·5	1	0·2
BMI (adults)				
Normal weight	4122	48·1	–	
Overweight	3296	38·5	–	
Obese	1151	13·4	–	
BMI (kids)				
Normal weight	–		422	82·9
Overweight/obese	–		87	17·1

Values are reported as number and percentages unless otherwise stated.

Beta-coefficients with 95 % confidence intervals (95 % CI) from multivariable-adjusted linear regression analysis were used to evaluate the association between socio-demographic characteristics, eating behaviours, psychosocial factors and dietary contribution of UPF (continuous-dependent variable).

Two models were ultimately fitted: Model 1 was adjusted for age, sex and energy intake, multivariable models 2 as in model 1 further adjusted for education, geographical area, place of residence, sport activity, occupation (adults), marital status (adults), smoking, BMI, CVD (adults), cancer (adults), hypertension (adults), diabetes (adults) and hyperlipidaemia (adults).

Missing data from categorical variables were assigned a missing indicator and were included in the models as dummy variables, similar to the way valid categories were represented.

Statistical hypotheses were tested using a two-tailed *P* < 0·05 level of significance.

Data analysis were generated using SAS/STAT software, version 9.4 (SAS Institute Inc.).

## Results

A total of 9078 participants (53·3 % women/girls) were included in the present study with a mean age of 54·5 years (age range 5–97 years; sd ± 17·2 years).

Study participants reported an average percentage of energy intake from UPF of 17·8 % (95 % CI (17·5, 18·1); IQR: 8·3–25·0; Table 1) and a mean UPF intake of 154·8 g/d (95 % CI (151·9, 157·7); IQR: 40·0–221·6). More than three-quarters (82·2 %) of total energies derive from unprocessed, minimally or moderately processed foods (see online Supplemental Fig. 1).

Distribution of daily energy intake according to four categories of the NOVA classification across main socio-demographic indicators is reported in Supplemental Fig. 1.

Processed meat (32·5 %), bread substitutes (16·7 %) and sweet biscuits (15·2 %) were the top contributing foods to the total UPF consumed in our study sample (see online Supplemental Table 2).

### Adult participants (20–97 years)

Among adults (*n* 8569), the average energy from UPF was of 17·3 % (95 % CI (17·1, 17·6); IQR: 8·0–24·4) for a total of 154·8 g/d of UPF eaten daily (95 % CI (147·5, 150·4); IQR: 40·0–211·2). Unprocessed or minimally processed foods provided nearly 40 % of total daily energies, processed foods an additional 24·4 % and processed culinary ingredients the remaining 19·6 % (Table 1).

The mean age of participants was 56·9 years (14·6), and they were prevalently women, residing in the Northern and Southern regions, with an average education and more frequently lived in pair and in urban areas (Table 1).

**Fig. 1:**
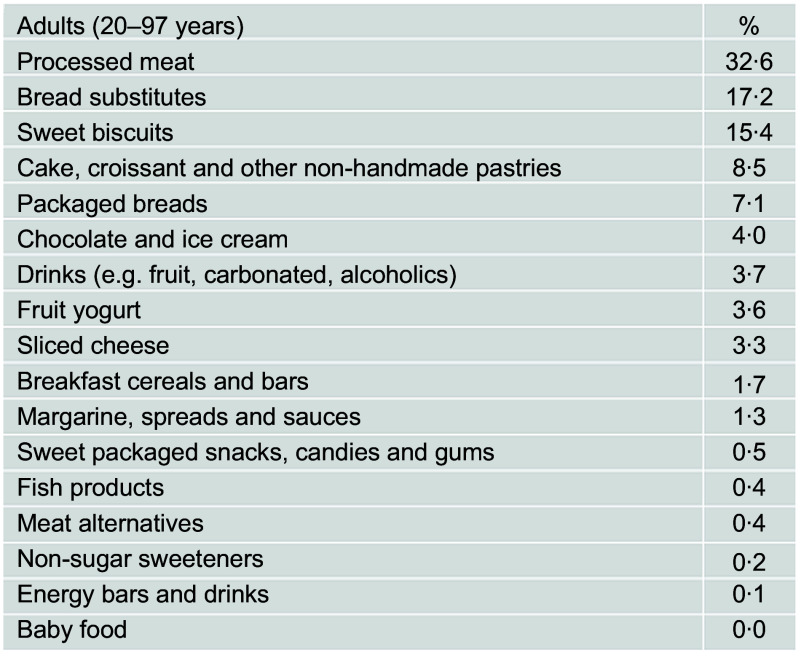
Contributing food groups (%) to the total amount of ultraprocessed food consumed among adults (*n* 8569) from the INHES study cohort, Italy 2010–2013

Processed meat (32·6 %), bread substitutes (17·2 %) and sweet biscuits (15·4 %) were the top contributing sources of total UPF eaten (Fig. 1).

Compared with the lowest (Q1), adult subjects in the highest quartile of UPF consumption (Q4) had higher intake of energy, sugar, protein, total fat, saturated fat, polyunsaturated fats, dietary cholesterol and Na, but lower intakes of total carbohydrate, fibre and monounsaturated fat (see online Supplemental Table 3).

Men (*β* = −1·28; 95 % CI (−1·89, −0·68); *β*s are dimensionless), older subjects (aged > 65 years *v*. 20–40 years; *β* = −3·10; 95 % CI (−4·40, −1·80)) and residents in Southern Italy (*v*. Northern Italy; *β* = −0·73; 95 % CI (−1·32, −0·14)) tended to consume less UPF as compared with their counterparts, while living in an urban environment was positively associated with UPF intake (*β* = 1·64; 95 % CI (0·87, 2·42); Table 2; Model 2).

**Table 2:** Demographic and socio-economic factors associated with ultra-processed food (UPF) intake in adults (20–97 years; *n* 8569) from the INHES study cohort, by means of adjusted regression coefficients (*β*) with 95 % confidence interval (95 % CI), Italy 2010–2013

	*n*	%	Ultra-processed food intake (% of total energy)							
			Crude mean	sd	*β*	95 % CI*	*P*-value	*β*	95 % CI†	*P*-value
Sex										
Women	4592	53·6	17·8	12·7	Ref.			Ref.		
Men	3977	46·4	16·8	12·2	−0·45	−0·75, −0·15	0·0029	−1·28	−1·89, −0·68	<0·0001
Age groups, years										
20–40	974	11·4	20·7	12·7	Ref.			Ref.		
41–65	4793	55·9	18·3	12·8	−2·12	−2·58, −1·66	<0·0001	−1·15	−2·14, −0·15	<0·0001
>65	2802	32·7	14·5	11·1	−4·08	−4·57, −3·58	<0·0001	−3·10	−4·40, −1·80	0·024
Geographical area										
Northern Italy	3500	40·9	17·8	12·9	Ref.			Ref.		
Central Italy	1391	16·2	17·5	11·6	−0·23	−0·99, 0·53	0·55	−0·23	−0·99, 0·53	0·56
Southern Italy	3678	42·9	16·8	12·3	−1·26	−1·83, −0·67	<0·0001	−0·73	−1·32, −0·14	0·014
Place of residence										
Rural	1168	13·6	15·5	11·8	Ref.			Ref.		
Urban	7401	86·4	17·6	12·5	2·11	1·36, 2·87	<0·0001	1·64	0·87, 2·42	<0·0001
Educational level										
Up, elementary	1532	17·8	14·6	11·1	Ref.			Ref.		
Lower secondary	2258	26·4	17·2	12·3	0·99	0·15, 1·84	0·021	0·70	−0·15, 1·55	0·11
Upper secondary	3413	39·8	18·0	12·5	1·23	0·41, 2·06	0·0034	0·55	−1·36, 0·74	0·20
Post-secondary	1375	16·0	18·7	13·6	1·57	0·60, 2·55	0·0016	0·65	−2·14, 0·44	0·22
Occupation										
Manual	1521	17·8	18·7	12·7	Ref.			Ref.		
Non-manual	2637	30·8	19·1	13·3	−0·16	−0·62, 0·94	0·69	−0·02	−0·85, 0·81	0·96
Housewife	952	11·1	17·3	12·1	−1·36	−2·43, −0·30	0·012	−0·79	−1·86, 0·29	0·15
Retired	3071	35·8	14·8	11·3	−2·09	−3·04, −1·15	<0·0001	−1·87	−2·83, −0·91	0·0001
Student	140	1·6	22·0	13·0	1·43	−0·81, 3·69	0·21	0·69	−1·60, 2·98	0·55
Unemployed	248	2·9	18·6	12·3	−0·54	−2·20, 1·11	0·52	−0·64	−2·30, 1·01	0·44
Marital status										
Married /in couple	6470	75·5	16·8	12·3	Ref.			Ref.		
Unmarried	1221	14·2	20·1	12·9	1·78	1·38, 3·50	<0·0001	1·26	0·37, 2·15	0·0053
Separated/divorced	272	3·2	19·9	14·4	2·47	0·98, 3·96	0·0011	1·88	0·38, 3·38	0·014
Widowed	606	7·1	15·9	11·9	0·94	−0·15, 2·02	0·090	1·16	0·07, 2·24	0·037
Smoking habit										
Non-smoker	5123	59·8	17·2	12·5	Ref.			Ref.		
Current	1374	16·0	18·0	13·3	0·30	−0·43, 1·04	0·43	0·21	−0·53, 0·95	0·58
Former	1909	22·3	17·1	11·9	0·78	0·11, 1·45	0·022	0·90	0·22, 1·57	0·0095
Occasional	163	1·9	17·5	12·1	−0·23	−2·15, 1·68	0·81	−0·50	−2·40, 1·41	0·61
Sport activity										
No	7010	81·8	16·8	12·2	Ref.			Ref.		
Yes	1559	18·2	19·8	13·2	2·11	1·43, 2·80	<0·0001	1·68	0·98, 2·38	<0·0001
CVD										
No	8283	96·7	17·3	12·5	Ref.			Ref.		
Yes	286	3·3	16·7	12·2	1·49	0·02, 2·96	0·047	1·64	0·16, 3·12	0·030
Cancer										
No	8280	96·6	17·3	12·5	Ref.			Ref.		
Yes	289	3·4	18·0	12·9	1·41	−0·03, 2·86	0·055	1·33	−0·11, 2·77	0·071
Hypertension‡										
No	5795	67·6	18·2	12·8	Ref.			Ref.		
Yes	2762	32·2	15·5	11·6	−1·09	−1·70, −0·49	0·0004	−0·61	−1·24, 0·02	0·059
Hyperlipidaemia‡										
No	6658	77·7	17·6	12·6	Ref.			Ref.		
Yes	1888	22·0	16·2	12·0	−0·41	−1·05, 0·23	0·21	−0·19	−0·85, 0·46	0·56
Diabetes‡										
No	7904	92·2	17·6	12·5	Ref.			Ref.		
Yes	644	7·5	14·5	11·2	−1·34	−2·35, −0·33	0·0092	−0·83	−1·85, 0·20	0·11
BMI										
Normal weight	4122	48·1	18·2	12·9	Ref.			Ref.		
Overweight	3296	38·5	16·6	12·0	−1·08	−1·89, −0·27	0·0090	−0·36	−0·95, 0·23	0·24
Obese	1151	13·4	16·4	12·0	−0·83	−1·41, −0·25	0·0053	−0·37	−1·22, 0·47	0·39

* Multivariable-adjusted linear regression including age groups, sex and energy intake (continuous).

† Multivariable-adjusted linear regression including all variables in the Table.

‡ Missing data: hypertension (*n* 12), hyperlipidaemia (*n* 23), diabetes (*n* 21).

Housewives and retired people were less likely to consume UPF as compared with manual workers (*β* = −0·79; 95 % CI (−1·86, −0·29) and *β* = −1·87; 95 % CI (−2·83, −0·91), respectively), while unmarried, separated and widowed reported higher UPF intake as opposed to those living in pair; among lifestyles, practicing sport activities and being former smokers were associated with higher consumption of UPF (Table 2; Model 2).

**Fig. 2:**
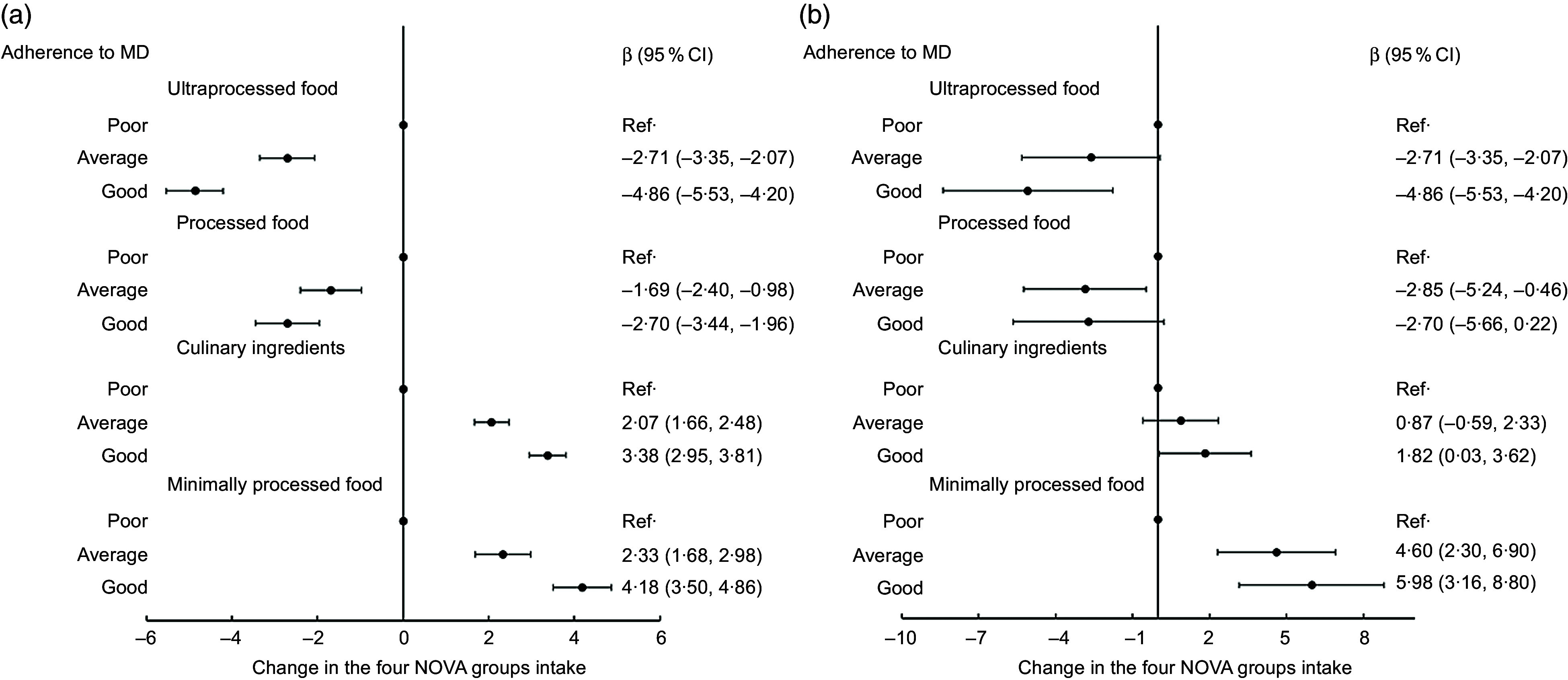
Contribution to total energy intake (%) of each food group according to its extent of processing (NOVA classification) by levels of adherence to the Mediterranean diet in (a) adults (20–97 years; *n* 8569) and (b) children/adolescents (5–19 years; *n* 509) from the INHES study cohort, Italy 2010–2013

Compared with poor adherence, good adherence to the Mediterranean diet was associated with a 4·86 % (95 % CI (4·20, 5·53)) lower energy intake from UPF (Fig. 2(a)).

Having breakfast and lunch out of home were associated with higher UPF intake, as compared with having these meals at home (*β* = 2·84; 95 % CI (1·46, 4·22); *β* = 3·14; 95 % CI (2·25, 4·04), respectively), and the same was true also for those individuals having meals while watching TV or PC; screen time (TV) was inversely associated with UPF intake, whereas snacking and frequent aperitifs were associated with more UPF eaten (Table 3).

**Table 3:** Association of meal patterns and eating behaviours with ultra-processed food intake in adults (20–97 years) and children/adolescents (5–19 years), by means of adjusted regression coefficients (*β*) with 95 % confidence interval (95 % CI), Italy 2010–2013

	Adults (*n* 8569)							Children/adolescents (*n* 509)						
	Ultra-processed food intake (% of total energy)							Ultra-processed food intake (% of total energy)						
	*n*	%	Crude mean	sd	*β*	95 % CI*	*P*-value	*n*	%	Crude mean	sd	*β*	95 % CI†	*P*-value
Breakfast														
Home	8018	93·6	17·2	12·3	Ref.			452	88·8	26·2	12·5	Ref.		
Out	320	3·7	21·1	13·7	2·84	1·46, 4·22	<0·0001	23	4·5	30·5	12·8	5·92	0·65, 11·18	0·028
Having breakfast														
Never/rarely	137	1·6	15·0	13·2	Ref.			41	8·1	19·5	14·1	Ref.		
Always	7576	88·4	17·7	12·3	3·24	1·18, 5·30	0·0020	456	89·6	26·5	12·4	6·34	2·27, 10·40	0·0023
Only with coffee	763	8·9	13·3	12·6	−1·23	−3·45, 0·98	0·28	9	1·8	25·8	14·4	8·53	−0·58, 17·63	0·066
Late morning snack														
Never/rarely	5958	69·5	16·7	12·4	Ref.			145	28·5	25·0	14·0	Ref.		
Always	2507	29·3	18·7	12·4	1·34	0·76, 1·92	<0·0001	360	70·7	26·2	12·1	0·14	−2·35, 2·62	0·91
Lunch														
Home	7528	87·9	16·8	12·2	Ref.			480	94·3	25·8	12·8	Ref.		
Out	908	10·6	21·6	13·8	3·14	2·25, 4·04	<0·0001	23	4·5	27·0	10·7	−2·82	−8·50, 2·85	0·33
Lunch														
Never/rarely	35	0·4	19·6	15·2	Ref.			1	0·2	31·2	–	Ref.		
Always	8446	98·6	17·3	12·4	−1·91	−5·97, 2·15	0·36	505	99·2	25·9	12·7	−6·55	−31·23, 18·13	0·60
Late afternoon snack														
Never/rarely	6293	73·4	16·7	12·3	Ref.			182	35·8	23·3	12·7	Ref.		
Always	2171	25·3	18·9	12·8	1·77	1·17, 2·38	<0·0001	323	63·5	27·5	12·4	3·33	0·99, 5·67	0·0054
Aperitif														
Never	7169	83·7	16·8	12·1	Ref.			426	83·2	26·4	12·6	Ref.		
Sometimes	1204	14·1	20·5	13·8	2·08	1·29, 2·86	<0·0001	45	8·8	24·6	12·9	−0·07	−4·16, 4·02	0·97
Always	129	1·5	19·8	13·9	1·17	−0·97, 3·32	0·28	35	6·9	22·1	13·3	−1·59	−6·32, 3·14	0·51
Dinner														
Home	8348	97·4	17·3	12·4	Ref.			495	97·3	26·0	12·7	Ref.		
Out	77	0·9	20·0	13·2	2·11	−0·64, 4·85	0·13	4	0·8	28·0	9·9	−0·37	−12·93, 12·19	0·95
Dinner														
Never/rarely	37	0·4	23·2	15·8	Ref.			1	0·2	26·5	–	Ref.		
Always	8435	98·4	17·3	12·4	−6·24	−10·17, −2·30	0·0019	505	99·2	25·9	12·7	2·30	−22·51, 27·11	0·86
Time spent watching TV														
<1 h/d	562	6·6	18·9	13·8	Ref.			50	9·8	25·6	11·8	Ref.		
≥1 < 2 h/d	2582	30·1	18·6	12·8	−0·58	−1·70, 0·54	0·31	189	37·1	25·4	12·9	−0·07	−4·07, 3·93	0·97
≥2 < 4 h/d	4305	50·2	16·9	12·2	−1·56	−2·66, −0·46	0·0053	249	48·9	26·4	12·6	0·34	−3·58, 4·27	0·86
≥4 h/d	1059	12·4	14·4	11·6	−2·71	−4·04, −1·38	<0·0001	16	3·1	25·0	13·0	−0·98	−8·12, 6·16	0·79
Time spent using PC														
<1 h/d	4485	52·3	15·9	11·9	Ref.			88	17·3	28·4	12·5	Ref.		
≥1 < 2 h/d	1606	18·7	19·9	13·1	1·63	0·83, 2·43	<0·0001	120	23·6	26·9	13·2	−0·11	−3·69, 3·44	0·95
≥2 < 4 h/d	1790	20·9	17·8	12·8	−0·55	−1·35, 0·25	0·18	208	40·9	25·4	12·1	−1·43	−4·85, 2·00	0·41
≥4 h/d	623	7·3	19·9	12·3	1·07	−0·07, 2·21	0·066	89	17·5	23·7	12·9	−2·39	−6·50, 1·73	0·25
Having meals while watching TV														
Never	7907	92·3	17·2	12·4	Ref.			460	90·4	26·0	12·7	Ref.		
Sometimes	525	6·1	19·0	12·5	1·87	0·79, 2·96	0·0007	35	6·9	25·6	12·2	−1·52	−5·91, 2·86	0·50
Most of the times	79	0·9	22·5	13·7	4·83	2·12, 7·53	0·0005	10	2·0	24·2	10·8	−1·13	−9·10, 6·84	0·78
Having meals while using PC														
Never	8394	98·0	17·3	12·4	Ref.			484	95·1	25·9	12·7	Ref.		
Sometimes	77	0·9	21·7	15·7	2·83	0·08, 5·57	0·044	19	3·7	26·9	11·4	−0·11	−5·97, 5·75	0·97
Most of the times	13	0·1	26·9	13·8	6·73	0·07, 13·38	0·048	2	0·4	33·4	13·7	7·55	−10·11, 25·22	0·40
Breakfast time														
Before 7:30 a.m.	4483	52·3	17·8	12·4	Ref.			317	62·3	26·5	12·5	Ref.		
After 7:30 a.m.	3763	43·9	16·7	12·5	−0·43	−0·98, 0·11	0·12	155	30·5	26·4	12·7	0·28	−2·19, 2·75	0·82
Lunch time														
Before 13:30 p.m.	5595	65·3	17·3	12·4	Ref.			135	26·5	28·5	13·4	Ref.		
After 13:30 p.m.	2974	34·7	17·5	12·6	−0·91	−1·51, −0·32	0·0027	374	73·5	24·9	12·2	−2·49	−5·35, 0·37	0·088
Dinner time														
Before 8:00 p.m.	3229	37·7	16·7	14·5	Ref.			116	22·8	27·4	13·8	Ref.		
After 8:00 p.m.	5340	62·3	17·7	12·4	0·11	−0·48, 0·71	0·71	393	77·2	25·4	12·3	−0·19	−3·13, 2·75	0·90

Numbers do not add up to 100 % due to missing values.

* For adults: multivariable-adjusted linear regression including age groups, sex, energy intake, geographical area, residence, educational level, occupation, marital status, smoking status, sport activity, CVD, cancer, hypertension, hypercholesterolaemia, diabetes and BMI.

† For children/adolescents: multivariable-adjusted linear regression including age groups, sex, energy intake, geographical area, residence, educational level, smoking status, sport activity and BMI.

Poor self-rated health status and reporting at least one adverse life event in the last year were associated with 5·32 % and 2·33 % higher contribution of UPF to total energy intake (95 % CI (2·66, 7·99) and 95 % CI (1·48, 3·18), respectively), as well as restless sleep (Table 4).

**Table 4:** Psychosocial factors associated with ultra-processed food (UPF) intake in adults (20–97 years; *n* 8569), by means of adjusted regression coefficients (*β*) with 95 % confidence interval (95 % CI), Italy 2010–2013

Psychosocial factors	*n*	%	Ultra-processed food intake (% of total energy)				
			Crude mean	sd	*β*	95 % CI*	*P*-value
Self-rated health status							
Excellent	1267	14·8	16·5	12·7	Ref.		–
Good	5569	65·0	17·7	12·5	2·86	2·08, 3·64	<0·0001
Fair	1586	18·5	16·8	12·3	3·63	2·63, 4·63	<0·0001
Poor	92	1·1	17·6	12·1	5·32	2·66, 7·99	<0·0001
Adverse life events							
None	7638	89·1	17·1	12·3	Ref.		–
At least one	931	10·9	19·1	14·0	2·33	1·48, 3·18	0·016
Stress at work							
Never	185	2·2	20·2	13·9	Ref.		
Sometimes/most of the times	3906	45·6	18·5	12·9	−2·98	−4·82, −1·13	0·0016
Often/always	488	5·7	20·4	13·6	−1·17	−3·28, 0·95	0·28
Not working/unascertained	3990	46·6	15·7	11·6	−3·47	−5·33, −1·62	0·0002
Stress at home							
Never	247	2·9	19·8	13·4	Ref.		
Sometimes	4759	55·5	17·0	12·4	−3·05	−4·62,-1·48	0·0001
Most of the times	3250	37·9	17·3	12·2	−2·95	−4·54, −1·37	0·0003
Often/always	299	3·5	20·9	14·0	0·55	−1·52, 2·61	0·60
Financial stress							
Little or none	184	2·2	18·6	13·0	Ref.		
Moderate	4879	56·9	18·8	12·6	−2·48	−4·28, −0·67	0·0071
High	3197	37·3	20·8	13·5	−4·10	−5·92, −2·28	<0·0001
Not working/unascertained	309	3·6	16·4	12·1	−1·67	−3·92, 0·58	0·15
Job control							
Little or none	530	6·2	20·3	13·3	Ref.		
Moderate/good	1234	14·4	17·9	12·6	−0·19	−1·44, 1·06	0·77
Very good	828	9·7	16·1	12·0	1·96	0·62, 3·29	0·0041
Not working/unascertained	5977	69·7	19·2	12·8	−0·96	−2·09, 0·17	0·097
Sleep quality							
Restful	7574	88·4	17·2	12·4	Ref.		
Restless	822	9·6	18·9	13·1	2·34	1·45, 3·23	<0·0001

Numbers do not add up to 100 % due to missing values.

* Multivariable-adjusted linear regression including age groups, sex, energy intake, geographical area, residence, educational level, occupation, marital status, smoking status, sport activity, CVD, cancer, hypertension, hypercholesterolaemia, diabetes and BMI.

Finally, all types of stress showed inverse associations with the consumption of UPF, while job control positively correlated (Table 4).

### Children/adolescents (5–19 years)

Among children/adolescents (5–19 years, *n* 509), the average energy from UPF was 25·9 % (95 % CI (24·8, 27·0); IQR: 17·0–34·1; Table 1), with a mean UPF intake of 277·6 g/d (95 % CI (259·5, 295·6); IQR: 125·1–381·0).

**Fig. 3:**
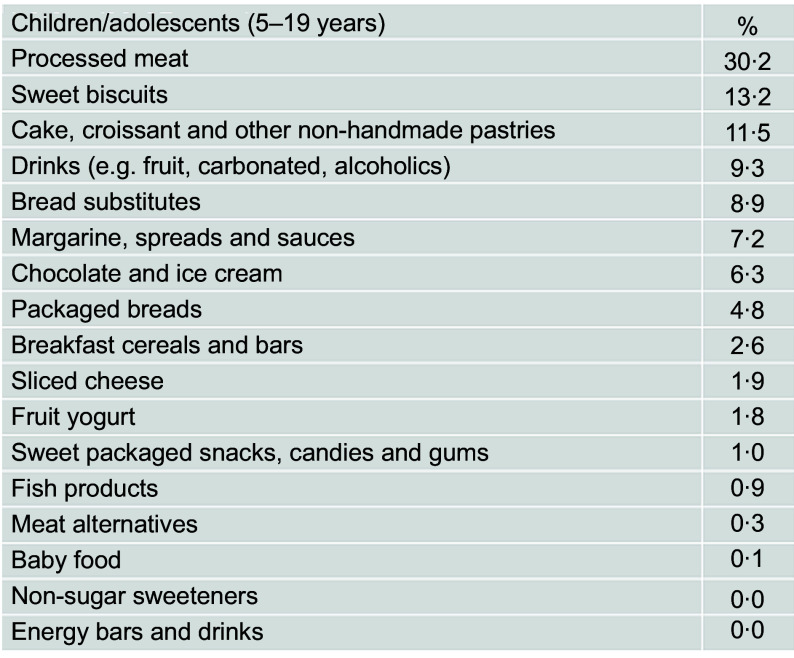
Contributing food groups (%) to the total amount of ultraprocessed food consumed among children/adolescents (*n* 509) from the INHES study cohort, Italy 2010–2013

Processed meat (30·2 %), sweet biscuits (13·2 %), cakes and other non-handmade pastries (11·5 %) and drinks (9·3 %) were the foods mostly contributing to the total of UPF consumed (Fig. 3). Nutrient characteristics of young participants across quartiles of UPF differed for sugar, Na and energy intake that were higher in those consuming excessive UPF and for total carbohydrates and fibre that were lower (see online Supplemental Table 4).

Boys consumed two-point percent more UPF of the total energy compared with girls (*β* = 2·01; 95 % CI (0·20, 3·82)), while adolescents and former/occasional smokers tended to consume less UPF products, as compared with their counterparts. Higher educational level was associated with reduced energies from UPF in the diet (*β* = −2·57; 95 % CI (−4·74, −0·40)) (Table 5; Model 2).

**Table 5:** Demographic and socio-economic factors associated with ultra-processed food (UPF) intake among children/adolescents (5–19 years; *n* 509) from the INHES study cohort, by means of adjusted regression coefficients (*β*) with 95 % confidence interval (95 % CI), Italy 2010–2013

	*n*	%	Ultra-processed food intake (% of total energy)							
			Crude mean	sd	*β*	95 % CI*	*P*-value	*β*	95 % CI†	*P*-value
Sex										
Girls	248	48·7	25·4	12·6	Ref.			Ref.		
Boys	248	51·3	26·4	12·7	0·69	−1·57, 2·95	0·55	2·01	0·20, 3·82	0·030
Age groups, years										
5–9 (children)	63	12·4	29·3	11·7	Ref.			Ref.		
10–19 (adolescents)	446	87·6	25·4	12·7	−4·13	−7·56, −0·70	0·018	−2·54	−3·56, 2·47	0·72
Geographical area										
Northern Italy	111	21·8	28·4	12·5	Ref.			Ref.		
Central Italy	44	8·6	25·5	12·8	−3·09	−7·56, 1·38	0·18	−0·79	−4·29, 2·72	0·66
Southern Italy	354	69·6	25·1	12·6	−2·94	−5·64, −0·23	0·033	−0·48	−2·68, 1·72	0·67
Place of residence										
Rural	66	13·0	26·2	13·9	Ref.			Ref.		
Urban	443	87·0	25·8	12·5	0·11	−3·20, 3·42	0·95	−0·55	−3·26, 2·14	0·69
Educational level										
Up to elementary	185	35·4	28·4	12·3	Ref.			Ref.		
Lower/upper secondary	324	63·6	24·5	12·7	−3·75	−6·40, −1·09	0·0058	−2·57	−4·74, −0·40	0·020
Smoking habit										
Non-smoker	451	88·6	26·4	12·7	Ref.			Ref.		
Current	40	7·9	22·4	11·0	−3·91	−8·05, 0·24	0·065	0·54	−2·74, 3·83	0·75
Former/occasional	18	3·5	19·8	12·4	−6·79	−12·75, −0·83	0·026	−4·99	−9·68, −0·29	0·037
Sport activity										
No	172	33·8	25·4	12·9	Ref.			Ref.		
Yes	337	66·2	26·1	12·5	0·72	−1·62, 3·07	0·54	−0·64	−2·54, 1·26	0·51
BMI										
Normal weight	422	82·9	25·8	12·4	Ref.			Ref.		
Overweight/obese	87	17·1	26·5	13·7	0·28	−2·70, 3·27	0·85	−1·80	−4·15, 0·56	0·13

* Multivariable-adjusted linear regression including age groups, sex and energy intake (continuous).

† Multivariable-adjusted linear regression including all the variables in the Table.

Good adherence to the Mediterranean diet was associated with five-point percent less energy from UPF as compared with poor (*β* = −5·08; 95 % CI (−8·38, −1·77)) and nearly 6 % higher energy from unprocessed/minimally processed food (*β* = 5·98; 95 % CI (3·16, 8·80) for good *v*. poor adherence) (Fig. 2(b)).

Having breakfast regularly and out of home positively correlated with UPF intake, as well as regular afternoon snacking, while early lunch time was inversely related (Table 3).

## Discussion

This study aimed to estimate the intake of UPF in a large cohort of 8569 adults and 509 children/adolescents residing in Italy and to investigate its major socio-demographic, psychosocial and behavioural correlates.

Average daily energies from UPF were 17·3 % and 25·9 % for adults and children/adolescents, respectively.

As expected, our UPF intake estimation in the adult sample was much lower than that reported in general populations from non-Mediterranean countries^(16–19)^, while being in line with data from another Mediterranean country such as Spain, where consumption of UPF was 24 % of the total energies eaten^(20)^.

Consistently, the percentage of UPF consumed by children/adolescents aged 5–19 years in our cohort tended to diverge substantially from data collected in the UK where 65 % of energies eaten by primary and secondary school children were from UPF^(33)^, and similar high dietary shares of UPF were documented in paediatric populations of the USA^(34)^ and Canada^(35)^.

Conversely, our estimations aligned with data from a Belgian cohort showing that the usual proportion of daily energy intake from UPF was 33·3 % for children and 29·2 % for adolescents^(36)^ and with those provided within the SENDO project in Spain^(37)^.

UPF was mainly consumed by urban residents, young people and those practicing physical exercise, a finding aligned with others^(11,20)^, possibly because active individuals tend to consume more frequently some highly processed foods, such as energy bars and drinks and health or slimming products.

As already documented in other populations^(38)^, higher intake of UPF was associated with lower diet quality, being richer in Na, fat and poor in fibre, and inversely with the Mediterranean diet, both in adults and children/adolescents, as previously seen by others^(11,37)^. Since the traditional Mediterranean diet features unprocessed or minimally processed food and emphasises home cooking, its inverse association with UPF possibly accounts for the relatively lower energy from UPF in our participants as compared with much higher estimations reported in other countries, especially non-Mediterranean.

Consistently, Southern Italian regions were likely to consume less UPF, possibly because highly processed foods may have more difficulty in establishing in a dietary context characterized by a strong food heritage as reflected by the Mediterranean diet^(39)^.

In the last decade, several population studies reported the adverse effects of UPF on health; indeed, excessive consumption of industrially processed food was associated with elevated risk of diet-related chronic disease^(9–12,14,15)^ and mortality^(10,13)^, also through mechanisms that include altered inflammation^(40)^.

Prospective studies following young children over time found that higher intake of UPF predicted higher total cholesterol, LDL cholesterol, tri-acyl glycerol and/or increased delta waist circumference^(6,41,42)^ and supported the role of UPF in the obesity epidemic in Brazilian adolescents and adults^(1)^.

Among potential mechanisms linking UPF consumption to increased health risk, chemicals largely used in the packaging of these food products, such as bisphenols and phthalates, were found to promote inﬂammation and oxidative stress^(43)^. In addition, food processing and particularly heat treatments produce neoformed contaminants (e.g. acrylamide) that are classified as genotoxic by the European Food Safety Agency^(44)^.

More recently, a study on 139 adolescents in Iran showed a significant urinary biomarker of DNA oxidative damage associated with higher intake of UPF^(45)^.

In the INHES cohort, both in adults and kids, processed meats were the top contributors to the dietary share of UPF, in accordance with estimations from the SUN cohort^(11)^ and the national Food Consumption Surveys in Belgium^(36)^, differently from adults our children/adolescents tended to consume more sugar-sweetened beverages in line with others^(36)^.

Among eating behaviours, regularly eating main meals out of home was associated with higher intake of UPF in adults, while for kids breakfast out of home was related to UPF intake suggesting that main foods included in the breakfast of Italian children are highly processed. Among adults, snacking likely led to consume more UPF, thus providing interesting suggestions on the type of foods preferably consumed by Italians between main meals.

Screen time while eating was directly associated with UPF intake in adults, a finding in accordance with prior evidence showing that TV watching possibly increases the amount eaten of high-density and palatable foods^(46)^, while in children and adolescents such association was not observed, likely because this behaviour in our young sample is underrepresented.

An important aspect of our research is the observed association between several aspects of the psychosocial dimension and their relationship with consumption of UPF. We found that higher consumption of UPF is associated with poor self-rated health status, a finding in line with prior epidemiological studies suggesting a direct relation between UPF intake and risk of depression^(14)^. We also found evidence of an association of adverse life events and UPF, in agreement with studies showing lower diet quality in stressed and neurotic individuals^(47)^. Finally, our findings revealed that participants reporting low sleep quality were more likely to consume UPF, in agreement with a recent study on 2499 Brazilian adolescents observing a direct relation between excessive UPF intake and poor sleep quality^(48)^.

### Strengths and limitations

This study has several strengths. First, results are from a large population sample of more than 9000 men and women recruited throughout Italy, with a complete assessment of diet, lifestyle and other covariates that minimise confounding. Also, our study shed light on correlates that, to date, had been less explored in other populations, such as meal patterns, eating behaviours and psychosocial factors.

Study limitations include the cross-sectional design and the telephone-based survey with potential interviewer bias and inability to use visual help. Also, the decline in the use of landlines may have resulted in an under-representation of respondents.

Furthermore, the study relies on self-reported health and dietary data that may lead to misreporting; however, data were collected by trained interviewers and each participant received by mail, beforehand, a short photograph atlas and guidance notes to estimate food portion sizes.

Confounding from unmeasured factors (e.g. additional psychological and socio-economic factors) cannot be fully ruled out. Also, dietary intakes were collected almost a decade ago, thus might not reflect the current UPF intake in the Italian population, although being the most updated data available so far, and in line with timeframes from the majority of studies in the field^(16–18)^.

However, the analyses on correlates of UPF intake are independent of the time of data collection and actually provide useful information for public health policies aimed to minimize the share of highly processed food in the diet.

Finally, the NOVA classification we used is still debated, mainly because of its equivocal definition of UPF and also because it has been revised and refined over time^(49)^; however, its usefulness in nutrition research has been widely acknowledged^(50)^.

In any case, some caution is needed in generalising these findings to other populations.

## Conclusions

Our results from a large cohort of adults and children/adolescents recruited in 2010–2013 throughout Italy showed that UPF constitutes less than 20 % of total energy intake among adults, while being approximately a quarter of the energies eaten by children/adolescents.

Such estimations are among the lowest recorded so far worldwide, but there is reason to believe that UPF intake is possibly on the rise as documented by worldwide trends^(36,51)^.

We did not observe substantial differences in UPF intake across socio-demographic strata, but rather identified some behavioural and psychosocial correlates that may promote increased UPF intake in the diet. In such, our findings provide relevant information to settle effective public health strategies at population levels by targeting groups at higher risk of unhealthy diets.

Finally, we observed that adherence to a Mediterranean diet, which features fresh or minimally processed foods and emphasises home cooking, possibly lowered the consumption of UPF in both adults and kids, thus potentially reducing the burden of major chronic diseases later in life.

Given the increasing dietary share of UPF worldwide and the growing epidemiological evidence on the adverse health effects of such foods, it is advisable to stress the importance of limiting UPF in dietary guidelines, as done in some^(52,53)^ but not in the majority of countries.

Food policy initiatives to minimise consumption of UPF in the diet primarily include fiscal measures, which, however, should be accompanied by subsidies or incentives (such as VAT reduction for healthful foods) aimed at promoting purchase of healthier foods; indeed, diets rich in UPF are estimated to be cheaper than diets with a low inclusion of these products; thus, the economical affordability of minimally processed foods might be favourably improved^(54)^.

Other actions would possibly include stricter regulations to reduce the advertisement and marketing of UPF, especially to children, front-of-package warning labels and targeted food policies to regulate access to and promotion of UPF in schools^(55)^.

## Supplementary material

For supplementary material accompanying this paper visit https://doi.org/10.1017/S1368980021002767. click here to view supplementary material

## References

[1] Louzada ML , Baraldi LG , Steele EM et al. (2015) Consumption of ultra-processed foods and obesity in Brazilian adolescents and adults. Prev Med 81, 9–15. 26231112 10.1016/j.ypmed.2015.07.018

[2] Monteiro CA , Moubarac JC , Cannon G et al. (2013) Ultra-processed products are becoming dominant in the global food system. Obes Rev 14, Suppl. 2, 21–28. 24102801 10.1111/obr.12107

[3] Monteiro CA , Cannon G , Moubarac JC et al. (2018) The UN decade of nutrition, the NOVA food classification and the trouble with ultra-processing. Public Health Nutr 21, 5–17. 28322183 10.1017/S1368980017000234 PMC10261019

[4] Monteiro CA , Cannon G , Levy R et al. (2016) NOVA. The star shines bright. Food classification. Public Health World Nutr 7, 28–38.

[5] Rauber F , Chang K , Vamos EP et al. (2020) Ultra-processed food consumption and risk of obesity: a prospective cohort study of UK Biobank. Eur J Nutr. Published online: 18 October 2020. doi: 10.1007/s00394-020-02367-1. PMC8137628 33070213

[6] Rauber F , Campagnolo PD , Hoffman DJ et al. (2015) Consumption of ultra-processed food products and its effects on children’s lipid profiles: a longitudinal study. Nutr Metab Cardiovasc Dis 25, 116–122. 25240690 10.1016/j.numecd.2014.08.001

[7] Scaranni PODS , Cardoso LO , Chor D et al. (2021) Ultra-processed foods, changes in blood pressure, and incidence of hypertension: results of Brazilian longitudinal study of adult health (ELSA-Brasil). Public Health Nutr 4, 1–9. 10.1017/S136898002100094X PMC10195295 33658095

[8] Levy RB , Rauber F , Chang K et al. (2020) Ultra-processed food consumption and type 2 diabetes incidence: a prospective cohort study. Clin Nutr 40, 3608–3614. 33388205 10.1016/j.clnu.2020.12.018

[9] Martínez Steele E , Juul F , Neri D et al. (2019) Dietary share of ultra-processed foods and metabolic syndrome in the US adult population. Prev Med 125, 40–48. 31077725 10.1016/j.ypmed.2019.05.004

[10] Kim H , Hu EA & Rebholz CM (2019) Ultra-processed food intake and mortality in the USA: results from the Third National Health and Nutrition Examination Survey (NHANES III, 1988–1994). Public Health Nutr 22, 1777–1785. 30789115 10.1017/S1368980018003890 PMC6554067

[11] Rico-Campà A , Martínez-González MA , Alvarez-Alvarez I et al. (2019) Association between consumption of ultra-processed foods and all-cause mortality: SUN prospective cohort study. BMJ 365, l1949. 31142450 10.1136/bmj.l1949 PMC6538973

[12] Fiolet T , Srour B , Sellem L et al. (2018) Consumption of ultra-processed foods and cancer risk: results from NutriNet-Santé prospective cohort. BMJ 360, k322. 29444771 10.1136/bmj.k322 PMC5811844

[13] Bonaccio M , Di Castelnuovo A , Costanzo S et al. (2021) Ultra-processed food consumption is associated with increased risk of all-cause and cardiovascular mortality in the Moli-sani study. Am J Clin Nutr 113, 446–455. 33333551 10.1093/ajcn/nqaa299

[14] Pagliai G , Dinu M , Madarena MP et al. (2021) Consumption of ultra-processed foods and health status: a systematic review and meta-analysis. Br J Nutr 14, 308–318. 10.1017/S0007114520002688 PMC7844609 32792031

[15] Lane MM , Davis JA , Beattie S et al. (2021) Ultraprocessed food and chronic noncommunicable diseases: a systematic review and meta-analysis of 43 observational studies. Obes Rev 22, e13146. 33167080 10.1111/obr.13146

[16] Baraldi LG , Martinez Steele E , Canella DS et al. (2018) Consumption of ultra-processed foods and associated sociodemographic factors in the USA between 2007 and 2012: evidence from a nationally representative cross-sectional study. BMJ Open 8, e020574. 10.1136/bmjopen-2017-020574 PMC5855172 29525772

[17] Rauber F , da Costa Louzada ML , Steele EM et al. (2018) Ultra-processed food consumption and chronic non-communicable diseases-related dietary nutrient profile in the UK (2008–2014). Nutrients 10, 587. 29747447 10.3390/nu10050587 PMC5986467

[18] Machado PP , Steele EM , Levy RB et al. (2019) Ultra-processed foods and recommended intake levels of nutrients linked to non-communicable diseases in Australia: evidence from a nationally representative cross-sectional study. BMJ Open 9, e029544. 10.1136/bmjopen-2019-029544 PMC6720475 31462476

[19] Polsky JY , Moubarac JC & Garriguet D (2020) Consumption of ultra-processed foods in Canada. Health Rep 31, 3–15. 10.25318/82-003-x202001100001-eng 33205938

[20] Blanco-Rojo R , Sandoval-Insausti H , López-Garcia E et al. (2019) Consumption of ultra-processed foods and mortality: a national prospective cohort in Spain. Mayo Clin Proc 94, 2178–2188. 31623843 10.1016/j.mayocp.2019.03.035

[21] Poti JM , Braga B & Qin B (2017) Ultra-processed food intake and obesity: what really matters for health-processing or nutrient content? Curr Obes Rep 6, 420–431. 29071481 10.1007/s13679-017-0285-4 PMC5787353

[22] Monteiro CA , Moubarac JC , Levy RB et al. (2018) Household availability of ultra-processed foods and obesity in nineteen European countries. Public Health Nutr 21, 18–26. 28714422 10.1017/S1368980017001379 PMC10260838

[23] Pounis G , Bonanni A , Ruggiero E et al. (2017) Food group consumption in an Italian population using the updated food classification system FoodEx2: results from the Italian Nutrition & HEalth Survey (INHES) study. Nutr Metab Cardiovasc Dis 27, 307–328. 28274729 10.1016/j.numecd.2017.01.004

[24] Giampaoli S , Palmieri L , Donfrancesco C et al. (2015) Cardiovascular health in Italy. Ten-year surveillance of cardiovascular diseases and risk factors: osservatorio epidemiologico cardiovascolare/health examination survey 1998–2012. Eur J Prev Cardiol 22, 9–37. 26195612 10.1177/2047487315589011

[25] Leclercq C , Arcella D , Piccinelli R et al. (2009) The Italian national food consumption survey INRAN-SCAI 2005–06: main results in terms of food consumption. Public Health Nutr 12, 2504–2532. 19278564 10.1017/S1368980009005035

[26] Illner AK , Harttig U , Tognon G et al. (2011) Feasibility of innovative dietary assessment in epidemiological studies using the approach of combining different assessment instruments. Public Health Nutr 14, 1055–1063. 21385523 10.1017/S1368980010003587

[27] Sette S , Le Donne C , Piccinelli R et al. (2011) The third Italian national food consumption survey, INRAN-SCAI 2005–06-part 1: nutrient intakes in Italy. Nutr Metab Cardiovasc Dis 21, 922–932. 20674305 10.1016/j.numecd.2010.03.001

[28] Trichopoulou A , Costacou T , Bamia C et al. (2003) Adherence to a Mediterranean diet and survival in a Greek population. N Engl J Med 348, 2599–2608. 12826634 10.1056/NEJMoa025039

[29] Serra-Majem L , Ribas L , Ngo J et al. (2004) Food, youth and the Mediterranean diet in Spain. Development of KIDMED, Mediterranean diet quality index in children and adolescents. Public Health Nutr 7, 931–935. 15482620 10.1079/phn2004556

[30] Rosengren A , Hawken S , Ounpuu S et al. (2004) Association of psychosocial risk factors with risk of acute myocardial infarction in 11119 cases and 13648 controls from 52 countries (the INTERHEART study): case-control study. Lancet 364, 953–962. 15364186 10.1016/S0140-6736(04)17019-0

[31] Italian National Institute of Statistics (2014) Statistical Atlas of Municipalities; available at https://www.istat.it/it/archivio/113712 (accessed September 2019).

[32] Cole TJ , Bellizzi MC , Flegal KM et al. (2000) Establishing a standard definition for child overweight and obesity worldwide: international survey. BMJ 320, 1240–1243. 10797032 10.1136/bmj.320.7244.1240 PMC27365

[33] Martines RM , Machado PP , Neri DA et al. (2019) Association between watching TV whilst eating and children’s consumption of ultraprocessed foods in United Kingdom. Matern Child Nutr 15, e12819. 30941879 10.1111/mcn.12819 PMC6859972

[34] Neri D , Martinez-Steele E , Monteiro CA et al. (2019) Consumption of ultra-processed foods and its association with added sugar content in the diets of US children, NHANES 2009–2014. Pediatr Obes 14, e12563. 31364315 10.1111/ijpo.12563

[35] Moubarac JC , Batal M , Louzada ML et al. (2017) Consumption of ultra-processed foods predicts diet quality in Canada. Appetite 108, 512–520. 27825941 10.1016/j.appet.2016.11.006

[36] Vandevijvere S , De Ridder K , Fiolet T et al. (2019) Consumption of ultra-processed food products and diet quality among children, adolescents and adults in Belgium. Eur J Nutr 58, 3267–3278. 30511164 10.1007/s00394-018-1870-3

[37] da Rocha BRS , Rico-Campà A , Romanos-Nanclares A et al. (2020) Adherence to Mediterranean diet is inversely associated with the consumption of ultra-processed foods among Spanish children: the SENDO project. Public Health Nutr 23, 1–10. 32698921 10.1017/S1368980020001524 PMC10195382

[38] Srour B , Fezeu LK , Kesse-Guyot E et al. (2019) Ultra-processed food intake and risk of cardiovascular disease: prospective cohort study (NutriNet-Santé). BMJ 365, l1451. 31142457 10.1136/bmj.l1451 PMC6538975

[39] Ruggiero E , Di Castelnuovo A , Costanzo S et al. (2019) Socioeconomic and psychosocial determinants of adherence to the Mediterranean diet in a general adult Italian population. Eur J Public Health 29, 328–335. 30020486 10.1093/eurpub/cky127

[40] da Silva A , Felício MB , Caldas APS et al. (2020) Pro-inflammatory diet is associated with a high number of cardiovascular events and ultra-processed foods consumption in patients in secondary care. Public Health Nutr 5, 1–10. 10.1017/S136898002000378X PMC10195276 33148359

[41] Costa CS , Rauber F , Leffa PS et al. (2019) Ultra-processed food consumption and its effects on anthropometric and glucose profile: a longitudinal study during childhood. Nutr Metab Cardiovasc Dis 29, 177–184. 30660687 10.1016/j.numecd.2018.11.003

[42] Leffa PS , Hoffman DJ , Rauber F et al. (2020) Longitudinal associations between ultra-processed foods and blood lipids in childhood. Br J Nutr 124, 341–348. 32248849 10.1017/S0007114520001233

[43] Ramadan M , Cooper B & Posnack NG (2020) Bisphenols and phthalates: plastic chemical exposures can contribute to adverse cardiovascular health outcomes. Birth Defects Res 112, 1362–1385. 32691967 10.1002/bdr2.1752 PMC7934580

[44] EFSA Panel on Contaminants in the Food Chain (2015) Acrylamide in food. EFSA J 13, 4104.

[45] Edalati S , Bagherzadeh F , Asghari Jafarabadi M et al. (2020) Higher ultra-processed food intake is associated with higher DNA damage in healthy adolescents. Br J Nutr 9, 1–29. 10.1017/S0007114520001981 32513316

[46] Blass EM , Anderson DR , Kirkorian HL et al. (2006) On the road to obesity: television viewing increases intake of high-density foods. Physiol Behav 88, 597–604. 16822530 10.1016/j.physbeh.2006.05.035

[47] Schweren LJS , Larsson H , Vinke PC et al. (2020) Diet quality, stress and common mental health problems: a cohort study of 121,008 adults. Clin Nutr 40, 901–906. 32654840 10.1016/j.clnu.2020.06.016

[48] Sousa RDS , Bragança MLBM , Oliveira BR et al. (2020) Association between the degree of processing of consumed foods and sleep quality in adolescents. Nutrients 12, 462. 32059416 10.3390/nu12020462 PMC7071336

[49] Gibney MJ (2018) Ultra-processed foods: definitions and policy issues. Curr Dev Nutr 3, nzy077. 30820487 10.1093/cdn/nzy077 PMC6389637

[50] Kelly B & Jacoby E (2018) Public health nutrition special issue on ultra-processed foods. Public Health Nutr 21, 1–4. 29227217 10.1017/S1368980017002853 PMC10260826

[51] Juul F & Hemmingsson E (2015) Trends in consumption of ultra-processed foods and obesity in Sweden between 1960 and 2010. Public Health Nutr 18, 3096–3107. 25804833 10.1017/S1368980015000506 PMC10277202

[52] Ministry of Health Brazil (2014) *Dietary Guidelines for the Brazilian Population*, 2nd ed.; available at http://189.28.128.100/dab/docs/portaldab/publicacoes/guia_alimentar_populacao_ingles.pdf (accessed April 2021).

[53] Food and Agriculture Organization of the United Nations (2017) Food-based dietary guidelines – Uruguay. http://www.fao.org/nutrition/education/food-based-dietary-guidelines/regions/countries/uruguay/en/ (accessed April 2021).

[54] Vandevijvere S , Pedroni C , De Ridder K et al. (2020) The cost of diets according to their caloric share of ultraprocessed and minimally processed foods in Belgium. Nutrients 12, 2787. 32933051 10.3390/nu12092787 PMC7551888

[55] Popkin BM , Barquera S , Corvalan C et al. (2021) Towards unified and impactful policies to reduce ultra-processed food consumption and promote healthier eating. Lancet Diabetes Endocrinol 9, 462–470. 33865500 10.1016/S2213-8587(21)00078-4 PMC8217149

