# Supplementary material for: Ultra-processed food consumption and its correlates among Italian children, adolescents and adults from the Italian Nutrition & Health Survey (INHES) cohort study
Source: Public Health Nutr. 2021 Jun 24;24(18):6258–71. doi: 10.1017/S1368980021002767 (PMC11148574; doi:10.1017/S1368980021002767)
Supplement: Supplementary file 1 [file S1368980021002767sup.zip › S1368980021002767sup002.doc]

**Supplemental Table 1.** Classification of food items in the INHES Study cohort according to the NOVA classification.

| **NOVA Food Category** | **Food Items** |
| --- | --- |
| **Group 1:** unprocessed or minimally processed foods | Water; fresh, squeezed or dried fruits and leafy and root vegetables; nuts; fresh legumes; wheat; rice; pasta; flour; potatoes; meat; poultry; fish and seafood; milk; plain yogurts without added sugar; eggs; spices; tea and coffee. |
| **Group 2**: processed culinary ingredients | Vinegars; creams; vegetable oils; butter; lard; sugar and honey. |
| **Group 3**: processed foods | Jam; cured traditional ham; olives; canned fruits; salted or sugared nuts; canned or bottled vegetables and legumes; breads; artisanal pizza; smoked and canned fish; cheese; wine and beer. |
| **Group 4**: ultra-processed food | Processed meat (e.g. salami, mortadella, sausages, hamburger, chicken nuggets); fish products (i.e. fish sticks); packaged breads and buns; bread substitutes (e.g. crackers, rusks, breadstick); breakfast cereals and bars; fruit yogurt; fruit drinks; carbonated soft drinks; cocoa drinks; alcoholic drinks (e.g. rum, gin, whisky); energy drinks and bars; milk substitutes (e.g. soy drinks); margarine; mayonnaise and similar; sliced cheese; sweet packaged snacks; plant-based meat alternatives (e.g. veggie burgers); non-sugar sweeteners; sweet biscuits; cakes, croissant and other non-handmade pastries; ice-cream; chocolate; candies and gums; non-sugar sweeteners; baby food. |
|  |  |

**Supplemental Figure 1.** Distribution of daily energy intake according to four categories of the NOVA classification across main sociodemographic indicators (n=9,078; 5-97 years) in the INHES Study cohort, Italy 2010-2013.

**Supplemental Table 2.** Contributing food groups (%) to the total amount of ultra-processed food consumed among participants (n=9,078; 5-97 years) in the INHES Study cohort, Italy 2010-2013.

| **Food group** | **%** |
| --- | --- |
| Processed meat | 32.5 |
| Bread substitutes | 16.7 |
| Sweet biscuits | 15.2 |
| Cake, croissant and other non-handmade pastries | 8.7 |
| Packaged breads | 6.9 |
| Chocolate and ice cream | 4.1 |
| Drinks (e.g. fruit, carbonated, alcoholics) | 4.1 |
| Fruit yogurt | 3.5 |
| Sliced cheese | 3.2 |
| Breakfast cereals and bars | 1.8 |
| Margarine, spreads and sauces | 1.7 |
| Sweet packaged snacks, candies and gums | 0.6 |
| Fish products | 0.4 |
| Meat alternatives | 0.4 |
| Non-sugar sweeteners | 0.2 |
| Energy bars and drinks | 0.0 |
| Baby foods | 0.0 |

**Supplemental Table 3.** Nutrient intake across quartiles of ultra-processed food consumption in the adult population (20-97 years; N=8,569) from the INHES Study cohort, Italy 2010-2013

|  | **Ultra-processed food consumption (% of total energy)** | | | | |
| --- | --- | --- | --- | --- | --- |
|  | Q1 | Q2 | Q3 | Q4 | P value |
| N of subjects (%) | 2,142 (25∙0) | 2,142 (25∙0) | 2,143 (25∙0) | 2,142 (25∙0) | - |
| Mean UPF (SD) | 3∙6 (2∙8) | 11∙6 (2∙1) | 19∙6 (2∙6) | 34∙5 (9∙0) | <0∙0001 |
| Total carbohydrates (%En) | 31∙8 (8∙6) | 30∙8 (8∙4) | 29∙6 (8∙3) | 27∙8 (8∙0) | <0∙0001 |
| Sugar (%En) | 14∙3 (6∙0) | 14∙9 (6∙1) | 15∙4 (6∙2) | 17∙2 (6∙6) | <0∙0001 |
| Protein (%En) | 15∙7 (3∙6) | 16∙2 (3∙7) | 16∙3 (3∙9) | 15∙8 (4∙0) | <0∙0001 |
| Total fat (%En) | 34∙1 (8∙1) | 34∙1 (7∙8) | 35∙0 (7∙6) | 35∙8 (7∙7) | <0∙0001 |
| Saturated fats (%En) | 9∙5 (3∙7) | 9∙5 (3∙6) | 10∙2 (3∙7) | 11∙3 (3∙8) | <0∙0001 |
| Monounsaturated fats (%En) | 18∙3 (5∙3) | 18∙0 (5∙1) | 18∙0 (4∙9) | 17∙2 (4∙9) | <0∙0001 |
| Polyunsaturated fats (%En) | 4∙0 (1∙5) | 4∙1 (1∙6) | 4∙2 (1∙6) | 4∙5 (1∙8) | <0∙0001 |
| Fiber (g/d) | 19∙3 (8∙3) | 18∙3 (8∙2) | 17∙6 (7∙7) | 16∙7 (7∙3) | <0∙0001 |
| Dietary cholesterol (mg/d) | 230∙4 (181∙1) | 229∙0 (168∙1) | 233∙9 (165∙0) | 246∙1 (161∙7) | 0∙0010 |
| Sodium (mg/d) | 1467∙8 (974∙9) | 1562∙9 (1071∙3) | 1610∙4 (1025∙7) | 1792∙8 (1069∙3) | <0∙0001 |
| Total energy (kcal/d) | 1852∙4 (563∙0) | 1955∙4 (598∙1) | 1991∙4 (589∙7) | 1951∙7 (591∙3) | <0∙0001 |

%En: percentage of total energy.

Means and p values adjusted for sex, age and energy intake.

**Supplemental Table 4.** Nutrient intake across quartiles of ultra-processed food consumption among children/adolescents (5-19 years; N=509) from the INHES Study cohort, Italy 2010-2013

|  | **Ultra-processed food consumption (% of total energy)** | | | | |
| --- | --- | --- | --- | --- | --- |
|  | Q1 | Q2 | Q3 | Q4 | P value |
| N of subjects (%) | 127 (25∙0) | 127 (25∙0) | 128 (25∙0) | 127 (25∙0) | - |
| Mean UPF (SD) | 10∙5 (5∙3) | 21∙2 (2∙4) | 29∙1 (2∙5) | 42∙6 (7∙3) | <0∙0001 |
| Total carbohydrates (%En) | 33∙8 (8∙1) | 32∙7 (7∙0) | 32∙4 (6∙9) | 28∙9 (7∙4) | <0∙0001 |
| Sugar (%En) | 12∙7 (5∙9) | 13∙8 (5∙4) | 14∙5 (5∙2) | 17∙5 (6∙1) | <0∙0001 |
| Protein (%En) | 16∙3 (3∙4) | 16∙6 (4∙0) | 16∙1 (3∙1) | 16∙0 (3∙4) | 0∙59 |
| Total fat (%En) | 36∙7 (7∙3) | 36∙5 (7∙3) | 36∙5 (6∙6) | 37∙3 (6∙8) | 0∙74 |
| Saturated fats (%En) | 10∙3 (3∙3) | 10∙6 (3∙6) | 10∙7 (3∙2) | 11∙4 (3∙6) | 0∙069 |
| Monounsaturated fats (%En) | 18∙7 (4∙4) | 18∙2 (4∙5) | 17∙9 (4∙3) | 17∙6 (3∙8) | 0∙19 |
| Polyunsaturated fats (%En) | 4∙9 (1∙6) | 4∙7 (1∙6) | 4∙8 (1∙4) | 5∙1 (2∙0) | 0∙37 |
| Fiber (g/d) | 17∙4 (8∙5) | 16∙1 (6∙4) | 15∙6 (5∙7) | 14∙6 (7∙1) | 0∙0023 |
| Dietary cholesterol (mg/d) | 301∙5 (215∙7) | 300∙6 (187∙1) | 268∙8 (133∙0) | 305∙1 (189∙7) | 0∙26 |
| Sodium (mg/d) | 1654∙2 (1057∙8) | 1774∙2 (1100∙0) | 1744∙7 (963∙5) | 2012∙2 (1386∙5) | 0∙027 |
| Total energy (kcal/d) | 2036∙6 (719∙1) | 2111∙4 (631∙2) | 2086∙2 (590∙8) | 2128∙6 (653∙3) | <0∙0001 |

%En: percentage of total energy.

Means and p values adjusted for sex, age and energy intake.
